# Deployment Strategies of Soil Monitoring WSN for Precision Agriculture Irrigation Scheduling in Rural Areas

**DOI:** 10.3390/s21051693

**Published:** 2021-03-01

**Authors:** Laura García, Lorena Parra, Jose M. Jimenez, Mar Parra, Jaime Lloret, Pedro V. Mauri, Pascal Lorenz

**Affiliations:** 1 Instituto de Investigación para la Gestión Integrada de Zonas Costeras, Universitat Politècnica de València, 46730 Grau de Gandia, Spain; laugarg2@teleco.upv.es (L.G.); loparbo@doctor.upv.es (L.P.); jojiher@dcom.upv.es (J.M.J.); maparbo@epsg.upv.es (M.P.); 2 Network and Telecommunication Research Group, University of Haute Alsace, 34 rue du Grillenbreit, 68008 Colmar, France; lorenz@ieee.org; 3 Instituto Madrileño de Investigación y Desarrollo Rural, Agrario y Alimentario (IMIDRA), Finca “El Encin”, A-2, Km 38, 2, 28800 Alcalá de Henares, Spain; pedro.mauri@madrid.org

**Keywords:** WSN deployment, orange orchards, WiFi, rural areas, ESP32, attenuation

## Abstract

Deploying wireless sensor networks (WSN) in rural environments such as agricultural fields may present some challenges that affect the communication between the nodes due to the vegetation. These challenges must be addressed when implementing precision agriculture (PA) systems that monitor the fields and estimate irrigation requirements with the gathered data. In this paper, different WSN deployment configurations for a soil monitoring PA system are studied to identify the effects of the rural environment on the signal and to identify the key aspects to consider when designing a PA wireless network. The PA system is described, providing the architecture, the node design, and the algorithm that determines the irrigation requirements. The testbed includes different types of vegetation and on-ground, near-ground, and above-ground ESP32 Wi-Fi node placements. The results of the testbed show high variability in densely vegetated areas. These results are analyzed to determine the theoretical maximum coverage for acceptable signal quality for each of the studied configurations. The best coverage was obtained for the near-ground deployment. Lastly, the aspects of the rural environment and the deployment that affect the signal such as node height, crop type, foliage density, or the form of irrigation are discussed.

## 1. Introduction

Agriculture is one of the fields in which the number of IoT solutions has increased the most [1,2]. These solutions consider many parameters that are necessary to improve and control the monitored crops so as to optimize irrigation and the use of additional nutrients and fertilizers. Therefore, varied sensors are deployed to monitor the soil; some aspects of the plants such as the amount of foliage, foliage color, plant height, or stem width; the amount of available water and water flow through the pipes; and some environmental parameters such as temperature and humidity. According to the type of crop to be monitored, the design of the IoT system may differ. For citrus plots in the Mediterranean coast of Spain, nodes may need to monitor soil, the quality, and the amount of water in the irrigation canals and the environment [3]. Monitoring nodes may also be mounted on vehicles such as drones or robots [4]. This monitoring process can be performed not only on the field, but it may also follow the produce to monitor its manufacturing process [5]. Furthermore, instead of just using one communication technology, two different wireless technologies may be used depending on the required communication distance, such as utilizing a gateway that receives the data from the sensors using WiFi and forwards it to the database using 3G. For precision agriculture systems that handle sensitive data, security solutions can be introduced to avoid the access of malicious third parties to the gathered data [6]. Lastly, the decision-making process that uses the monitored information to determine the actions that the actuators must execute has become easier. Artificial intelligence (AI) is employed to process the data to provide recommendations and perform predictions [7]. For the case of intelligent irrigation systems for smart agriculture, data on temperature and humidity of the air and the soil can be analyzed to determine when to start and stop irrigation to optimize water usage.

Automating the irrigation schedule is one of the main tasks of the PA systems. This is often performed with the use of agrometeorological data from meteorological stations that can be placed far from the fields. The introduction of soil monitoring nodes has led to the use of soil variables to make scheduling more precise. The water balance formula has been adjusted with data from soil moisture sensors to implement regulated deficit irrigation (RDI) strategies [8]. A homogenization of the production was achieved after applying the RDI strategies with the automatic irrigation system. The Van Genutchen model has also been utilized by some irrigation scheduling systems to determine moisture usage and obtain water savings between 56.4% and 90% [9]. Predictive irrigation scheduling systems have been developed as well, utilizing AI and machine learning techniques. A data-driven robust model predictive control (DDRMPC) for irrigation needs prediction utilized learning-based techniques to create an uncertainty set obtained from existing data logs [10]. The results of applying DDRMPC showed a reduction of 40% in water consumption. Machine learning has also been employed to calculate the reference evapotranspiration (ETo) of the crop based on the data from soil moisture sensors, obtaining the best results with the randomizable filtered classifier technique followed by artificial neural networks [11]. Lastly, performing climate modelling [12] could also be utilized to predict the resource requirements of a crop.

The lack of energy supplies is another one of the main challenges to consider when implementing IoT systems for agriculture. Since power supplies are scarce in many locations in rural areas, powering the IoT devices deployed on the field is generally done using batteries and solar panels. Therefore, small-sized IoT devices with low energy consumption are usually employed for both data acquisition and actuator management. Moreover, low-power protocols can be utilized as well so as to reduce energy consumption as much as possible [13,14]. 

Due to the aforementioned reasons, implementing feasible and appropriate solutions for smart agriculture presents many challenges [15]. However, the amount of data may not be one of them. The amount of transmitted data for smart agriculture WSN is usually small, as the monitored parameters usually change at a slow pace. However, the consistent transmission of the data is necessary. Furthermore, the number of IoT devices deployed in the field must be minimized with the aim of reducing the cost while guaranteeing the performance of the system. One of the ways of minimizing the number of nodes is deploying the nodes at the maximum possible transmission distance for the selected wireless technology. However, the maximum range between the nodes may vary depending on the type of crop planted on the monitored field. 

The coverage of the IoT devices deployed on the field may vary greatly based on the selected wireless technology, the height at where the devices are located, and other factors regarding signal quality such as possible interferences, distance attenuation, absorptions, reflections, dispersion, or the multipath effect. Therefore, the effective range achieved by IoT devices deployed at crops can be considerably reduced. It is, therefore, necessary to know the limitations regarding the range of the devices when implementing a solution for agriculture so as to design the network according to the characteristics of the environment. The received signal strength indicator (RSSI) allows obtaining a measure of the quality of the wireless signal and can be utilized for the localization of the devices [16,17]. Although there are varied metrics utilized for estimating link quality, RSSI is a good measure when the focus is on the coverage of a wireless connection and assessing the quality of the link that can be obtained establishing ranges that indicate if the connection is good or bad. For grass fields, the threshold that was established to indicate poor quality links was −90 dBm [18]. Authors in [18] also determined that no conclusion has been reached on whether RSSI or LQI (link quality indicator) is better than the other, as there are contradictory results and remarks in the literature. They state that each metric is able to provide information on some aspects regarding the quality of the link. However, there is no commonly used metric that is able to provide information on all available aspects regarding link quality unless it is a newly proposed metric, which has, in turn, a big overhead. Therefore, RSSI can be considered a good enough metric for the purpose of performing coverage studies.

As there is not a study performed on every type of crop for every deployment necessity, an identification of the key aspects that influence PA WSN deployments for soil monitoring purposes would provide useful insights. In this paper, different deployment configurations for the presented PA system are studied with the focus on orange orchards, and other types of vegetation with different height and foliage density such as grasslands and scrub fields. The key aspects that affect a PA WSN design are identified by determining the effects of the rural environment on the signal. The description of the soil monitoring nodes, the architecture of the PA system, and the irrigation algorithm that utilizes the gathered data to estimate irrigation requirements are provided as well. The testbed includes on-ground, near-ground, and above-ground deployment strategies. Furthermore, the tests were performed with low-cost ESP32 Wi-Fi nodes placed inside protective cases. The analysis of the testbed results has been done to determine the theoretical maximum coverage distance with acceptable signal quality for each of the deployment configurations. Lastly, the challenges presented by the type of vegetation, the foliage density, or the form of irrigation, as well as factors such as node height, energy consumption, or node density for PA WSN deployments are discussed. The main contributions of this paper are summarized as:

- A soil monitoring proposal including an algorithm to determine the irrigation needs based on FAO recommendations and the sensed data.
- Different deployment strategies for low-cost soil monitoring nodes have been tested on real environments for three different vegetation types: orange orchards, scrublands, and grasslands.
- Finally, the key aspects of deployments of soil monitoring nodes and the results obtained from the performed tests have been discussed.

The rest of the paper is organized as follows. Section 2 presents the related work. Section 3 describes the materials and methods utilized for this study. The results are depicted in Section 4. The discussion and challenges are presented in Section 5. Finally, the conclusion and future work are presented in Section 6.

## 2. Related Work

WiFi is a widely spread wireless communication technology. Many agricultural monitoring systems use WiFi for their communication between the different agents of their architectures, such as the one proposed in [19] by Gerard Rudolph Mendez et al. They presented a WiFI wireless sensor network for agriculture monitoring where temperature, humidity light, soil moisture, and water level data are collected by the nodes. Furthermore, the data were forwarded to a server so they could be accessed later. Therefore, studying the coverage of WiFi in different agricultural environments is of great interest. Moreover, Muhammad A. et al. presented in [20] the location estimation of wireless nodes utilizing signal strength. Urban areas, rural areas, forests, and plantations were the utilized locations, and the IEEE 802.11 b/g standard was employed. The experiment was repeated five times and the mean value was obtained. The error rate was obtained for each terrain, resulting in the minimization of the error between 3 and 18.5 m according to the terrain.

The effects of vegetation on radio signals have been discussed by and analyzed by other authors. Jose Antonio Gay-Fernández et al. performed in [21] wireless propagation and path loss modeling for environments with vegetation for the 2.4, 3.5, and 5.5 GHz frequencies in a peer-to-peer configuration. Tests were performed in different environments, where the results showed better propagation models for grasslands followed by forests and scrublands. However, the authors remarked the importance of vegetation density. The attenuation caused by the foliage of tropical vegetation for frequencies 2 to 18 GHz and 26.5 to 40 GHz was assessed by Hairani Maisarah Rahim et al. in [22]. The results showed that the obtained attenuation depended on the frequency with results varying from 10.99 dB at 15 GHz to 24.23 dB at 9 GHz. J. Acuña et al. presented in [23] a study on the interferences caused by vegetation barriers to wireless networks with the aim to reduce the signal strength at the areas where coverage is not wanted. Several species of shrubs with different dimensions were studied. Results showed attenuations reaching 10 dB for 2.4 GHz and 21 dB for 5.8 GHz. Leire Azpilicueta et al. performed in [24] a studio on the propagation of radio waves at the 2.4 GHz band through inhomogeneous vegetation environments. Measures were simulated in a park environment with grass, trees, and concrete as the elements that introduce attenuation using ZigBee and Bluetooth wireless technologies. The experimental measures were performed with Zigbee mote, which was placed at the trunk of a tree. The results verified that their proposed 3D ray launching algorithm was good enough for radioplanning in such environments. A model for radiowave propagation through the foliage of different tree species and locations for frequencies of 1 to 60 GHz was presented by Jürgen Richter et al. [25]. Results showed that the estimated RMS error was 8.38 dB compared to the 11.51 dB of the ITU-R 833-3 model. A study of wideband signal propagation from 1 to 60 GHz through different types of trees was performed by D. L. Ndzi et al. in [26]. The signal power is obtained with an omnidirectional antenna. Results showed that the signal path depends on the width and height of the vegetation. A simulation of the effects of vegetation on radiowave propagation for WSN was performed by Naseer Sabri et al. in [27]. The authors utilized the free space path loss model and added foliage models to simulate the attenuation. The simulations were performed for frequencies from 1 to 3 GHz, distances up to 20 m, and heights between 0.5 and 2 m for the receiver and 3.5 m for the transmitter.

The factors that create signal attenuation in wireless transmissions through vegetation at 1.3, 2, and 11.6 GHz were discussed by Nick Savage et al. [28]. The maximum attenuation (MA), the nonzero gradient (NZG), and modified exponential decay (MED) were utilized to model the attenuation caused by vegetation. A wideband channel sounder was utilized to perform the measures at heights varying from 2.5 to 7.5 m. Results showed that vegetation density, the measurement geometry, and the state of the leaf are the factors that most contribute to the attenuation. The 11.6 GHz frequency was the one that presented a greater attenuation due to the leaves. A study on the radio wave propagation of 433 MHz signals at potato fields utilizing RSSI measures was performed by John Thelen et al. [29]. Results showed a communication range of 78 m and advise to place nodes between a 23 m range when the crop is on its return and a 10 m range when the crop is flowering. Furthermore, a characterization of vegetation movements on radiowave propagation was performed by M. H. Hashim et al. in [30]. The measures were taken in an anechoic chamber at 0.9, 2, 12, and 17 GHz with two types of trees and four different settings for wind. The results showed that the behavior of the signal propagation varied with calm and windy conditions, but it did not vary much among different windy conditions. 

Lastly, other studies have performed different tests of smart sensing deployments on outdoor environments to determine the effects of different types of obstacles and vegetation on the received signal. A remote sensing WSN framework for smart city monitoring was designed by Ala’ Khalifehet al. [31]. Tests were performed using ZigBee in outdoor locations with varied obstacles. The results showed differences between the coverage advertised by the manufacturer and the measured signal strength. A communication protocol was proposed as well to enable the communication between ground nodes and a UAV. The authors stress the importance of performing preliminary tests before designing and deploying the network. A smart irrigation system for urban areas that utilized LoRa was presented by Iván Froiz-Míguez et al. [32]. Air temperature and soil moisture and temperature were monitored to determine water requirements. The authors modeled the campus area with varied obstacles for both 868 and 433 MHz transceivers performing near-ground and underground communication. The results showed low propagation losses for the 433 MHz band. Furthermore, sufficient coverage was obtained even for the worst gateway location. RSSI measures of IEEE 802.15.4 low-cost radio transceivers were performed by Jan Bauer et al. [33] to determine vegetation growth. Experiments were performed on wheat fields. Moreover, the effects of different meteorological variables are studied. The results showed that temperature and absolute humidity are the main factors that affect the communication. However, other meteorological variables did not present any influence. Furthermore, ground sensors were strongly affected by vegetation.

In this paper, a PA system that utilizes weather and soil data from low-cost sensors to calculate irrigation requirements is proposed. Furthermore, we study the RSSI provided by WiFi APs implemented with ESP32 microcontrollers, which are highly used for agricultural IoT monitoring applications for different vegetation types and deployment configurations. The novelty of this study relies on its focus on WSN deployment strategies specific for PA applications, where different configurations of node placements are tested with vegetation of varied characteristics, expanding the existing knowledge (see Table 1). Furthermore, specific challenges to PA wireless networks are discussed.

## 3. Materials and Methods

This section presents a background on soil monitoring in precision agriculture, the proposed architecture, the proposed irrigation scheduling algorithm, and the description of the testbed. 

### 3.1. Soil Monitoring Background

The state of the soil is critical in agriculture. Monitoring known parameters that characterize the soil is key for precision agriculture systems, as they are needed to determine the amount of water for the irrigation of the crops and to optimize the production. The available sensors to monitor the characteristics of the soil can perform their measurements through the physical characteristics of the soil or its chemical characteristics. However, chemical sensors are not adequate for these systems, as they would require the assistance of a person to clean, calibrate, and perform the measurement. Therefore, a precision agriculture system will usually be comprised of sensors that need low maintenance. 

Soil quality depends on several aspects, and its degradation is mostly caused by soil compaction, acidification, and salinization. The salinity, acidity, and water holding capacity of the soil are some of the most considered aspects when monitoring the state of the soil, but other factors such as nutrient availability, labile and organic carbon, rooting depth, and soil structure and texture affect the quality of the soil as well [45].

Most precision agriculture systems focus then on three factors to characterize the soil. The PH of the soil is measured to determine its acidity or alkalinity. Very acid soils can lead to deficiencies in nutrient availability despite the addition of fertilizer. Low PH levels can result in aluminum toxicity leading to poor growth, smaller quantity, and produce size. Moreover, the persistence of herbicides can be affected by the PH levels as well. Lastly, microbial activity can be affected as well leading to unhealthy plant conditions.

Soil humidity is measured to determine if the crops are suffering from water stress due to a lack of water in the soil. High humidity results in root diseases and the waste of irrigation water. On the other hand, low humidity can cause yield loss and even the death of the plant. Furthermore, the movement of the water in the soil determines how the nutrients will reach the plants, whether they are added to the irrigation water or through other means. The content of clay, sand, and silt of the soil determines its water holding capacity.

Soil temperature experiments change within the day and throughout the year. Solar radiation and air temperature are determinant factors on the temperature of the soil. Soil temperature can affect the chemical and physical properties of the soil. The PH increases with soil temperatures between 25 and 39 °C [46]. The moisture content, salinity, and the structure and aeration of the soil are affected as well. The organisms and the organic matter in the soil are influenced by soil temperature, resulting in more soil respiration between temperatures of 10 and 28 °C, an increase in the activity of micro-organisms and macro-organisms between temperatures from 10 to 24 °C, and the decomposition of the organic matter with temperatures between 2 and 38 °C. Furthermore, the water uptake of the plants gets reduced with high soil temperatures and the nutrient uptake decreases with low soil temperatures. Lastly, high soil temperatures lead to an improvement in root growth, whereas low soil temperatures result in a decrease in root growth.

## 3.2. Architecture Description

**Figure 1:**
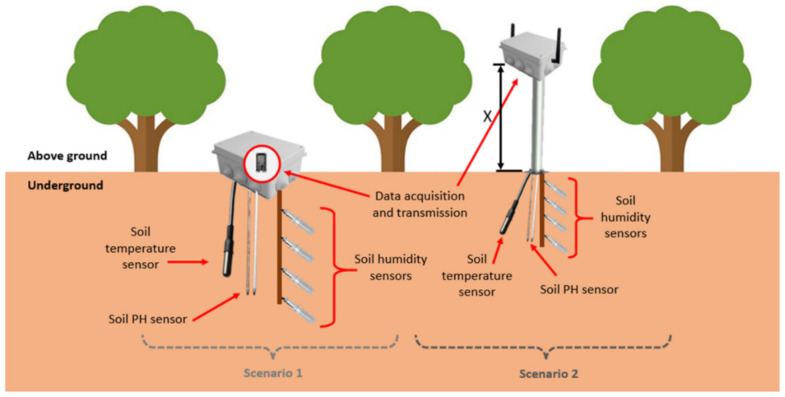
Proposed soil monitoring node for scenarios 1 and 2.

Our proposed soil monitoring system is presented in Figure 1. The data acquisition and transmission are performed with the ESP 32 Devkit v1 node. Although this node is not an industrial node, the low-cost nodes can also be utilized in realistic agricultural environments, considering real-time data transmission is not necessary for the proposed system. Furthermore, the node is placed inside a protective box to avoid damage from the weather, animals, or machinery. Utilizing low-cost nodes and sensors in precision agriculture solutions also allows farmers to improve the performance of their crops and optimize resources without investing large amounts of money, which makes the proposed solution more accessible. Temperature is measured with a soil temperature monitoring sensor probe such as the THERM200 [47]. The soil humidity multi-sensor array is comprised of low-cost soil humidity sensors such as the FS200-SHT10 sensor [48] at depths of 20, 30, 40, and 50 cm in a manner similar to that presented in [49] for the scenario of a sensor buoy. Lastly, a low-cost soil PH sensor is connected to the node to monitor the PH of the soil as the MS02 [50]. The probes are inserted in the soil to gather the measurement. These probes act as a fixing structure to ensure that the box does not move due to the meteorological conditions, mainly wind and rain, or due to the passing of fauna. Moreover, due to the general characteristics of citrus orchards, we do not expect high soil erosion, which might alter the location of the sensor or the generation of disturbances in our measures. 

As it is further described in Section 3.4, two scenarios are contemplated, one with the node placed in an on-ground position and the other with the node located above-ground at different heights. The tests have been performed considering a deployment strategy that is focused on the field area, where the nodes are located among the crops both on the surface of the soil and above the ground, contemplating that the sensors must be buried into the ground. There are other areas in PA systems such as the canals where the irrigation water is transported that can be monitored with sensors such as the conductivity sensor presented in [51]. However, they are not the focus of this paper.

**Figure 2:**
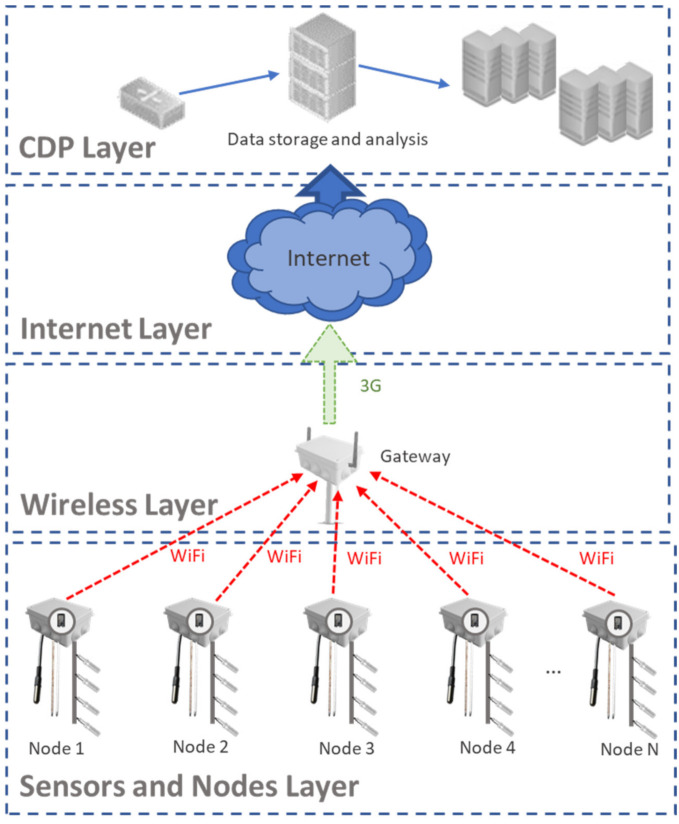
Architecture.

Figure 2 shows the four layers that form the proposed architecture where the ESP32 nodes are located. The ESP32 nodes are located in the lower layer, which have the necessary sensors connected to them to obtain the information from the environment. The characteristics of the utilized nodes and their antenna are depicted in the specifications of the nodes in [52,53]. These nodes perform the transmission using the IEEE 802.11 standard, with a maximum data rate of 150 Mbps in the 2.4 GHz band. Their low cost makes their use in agricultural systems affordable to farmers. Furthermore, power consumption can be optimized by programming the nodes to go to sleep mode when measures are not being taken. Moreover, these applications do not usually record video or transmit data in real-time, as variations in the state of the crops are slow, resulting in a reduced amount of data for transmission [3]. In the immediate upper layer, a network of wireless access points (APs) is available, through which the data are sent to an AP that acts as a gateway to the Internet. That gateway receives the data from the node and uses 3G to forward the data to the database. The Internet is in the next layer. Finally, the upper layer is where the data is stored, and its consultation and treatment of the obtained data is performed. The farmers can access the data through a web or a mobile interface. 

## 3.3. Proposed Algorithm

In this subsection, we depict the performance algorithm of the irrigation system.

**Figure 3:**
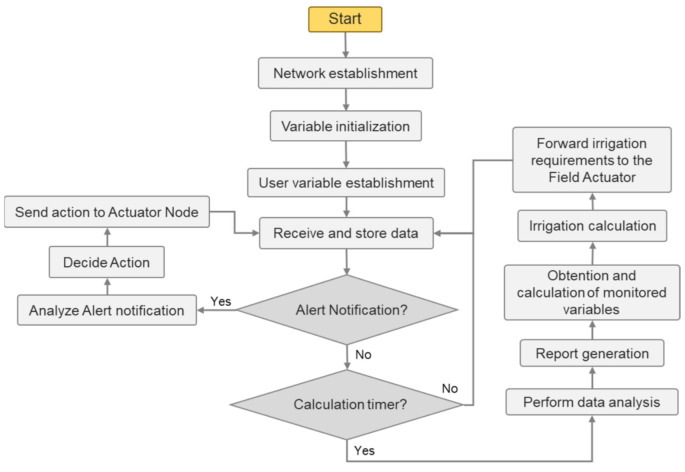
Flow chart of the Data Center.

The presented system determines the irrigation requirements of the fields according to the weather and the state of the soil. For the field area, an irrigation software was created to analyze the data from the fields at the Data Center so as to determine the amount of water required by the fields. The flow chart of the performance of the Data Center is presented in Figure 3. After the network establishment, all the variables, fixed and obtained from both the user and the sensors, are initialized. All the required variables are listed in Table 2. Then, the Data Center receives all the data from the sensors and stores them for later analysis. The system generates an alert if some variables such as the salinity of the soil or the water surpass a threshold. Therefore, if the data center receives any Alert Notification, the notification is processed, and then an Action is decided and forwarded to the relevant actuator. If there is no alert notification, the system checks if the calculation timer is reached. The calculations for the irrigation requirements need to be performed once a day as the irrigation is done each morning. Therefore, when that time is reached, all the data are processed to create reports for the user. Then, the intermediate variables are calculated. Lastly, the calculated irrigation requirements are forwarded to the Field Actuator for it to irrigate the fields the following morning. The PA system may include irrigation water monitoring and an agrometeorological monitoring node to obtain more precise irrigation schedules for the intended fields. However, this paper is focused on the field area.

Algorithm 1 presents the algorithm that determines the irrigation needs of the crops. The basis of the calculations of the irrigation requirement of a crop is presented on the FAO Irrigation and Drainage Paper No. 56 on Crop Evapotranspiration [54]. For our system, we introduced the monitored parameters as variables of the equations for irrigation calculation presented by the FAO. Although the FAO does not contemplate the use of sensors deployed on the field to acquire the necessary data for the calculations of the evapotranspiration of the crop (ETc), many papers have implemented varied forms of ETo (reference evapotranspiration) and ETc calculations using the data from sensors deployed on the fields [9,10,55]. Algorithm 1 is executed once a day after the data center receives the data gathered throughout the day. This is done because some variables require the minimum and maximum value of a day to perform the calculations, such as temperature and relative humidity, and other variables require a total of the day, such as sunlight hours and precipitation amount. Therefore, the monitoring process and the reception of the data at the specified intervals are contemplated in the flow chart in Figure 3, whereas Algorithm 1 receives the processed data of the day with the variable initialization process, as well as the fixed variables. Then, the variables set by the user are initialized. Using all the gathered data, the ETo is calculated. Then, the crop stage is determined. The crop stage differs among crop types and the time of the year. Then, the presence of water stress and salinity stress is determined according to the readings from the soil sensors so as to adjust the irrigation requirements as stated in the recommendations of the FAO. Water stress and salinity stress can result in higher water requirements in an effort to counteract the caused damage. Furthermore, if precipitation is detected, the amount of irrigation water may be reduced or the irrigation day may be postponed according to the amount of precipitation. This is calculated utilizing the irrigation adjustment due to precipitation as recommended by the FAO. Lastly, the ETC is calculated and the irrigation requirements are determined.

**Algorithm 1.** Irrigation Algorithm.1Variable initialization2User parameter initialization3ETo calculation4Determination of the Crop Stage5**If** Water stress **then**6   Calculate irrigation adjustment due to water stress7
**end if**
8**If** High salinity levels **then**9   Calculate irrigation adjustment due to salinity10
**end if**
11**If** Precipitation **then**12   Determine the precipitation amount13   Determine the hour of the precipitation14   Calculate irrigation adjustment due to precipitation15
**end if**
16Calculate ETc 17Calculate Irrigation requirements of the crop18**End**.

**Figure 4:**
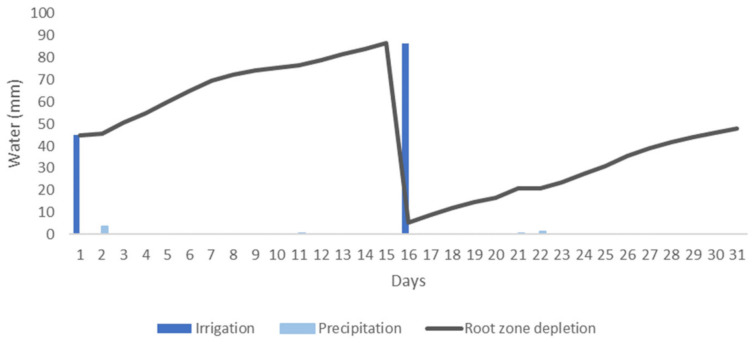
Simulation of an irrigation schedule for an orange field in Murcia.

Figure 4 presents the case of an irrigation schedule for an orange orchard in a Mediterranean climate for one month. The climate data were obtained from the agrometeorological station MO12 in Región de Murcia, Spain [56], for the month of October 2020. During the period of one month, there were four precipitation events and one water stress event. As it can be seen, the irrigation scheduling begins with one initial irrigation event. Then, the root zone depletion is calculated to determine the irrigation needs of the orange trees. Water stress is detected on day 15 and another irrigation event is scheduled for day 16. It is noticed that the root zone depletion is reduced by both irrigation and precipitation events.

**Figure 5:**
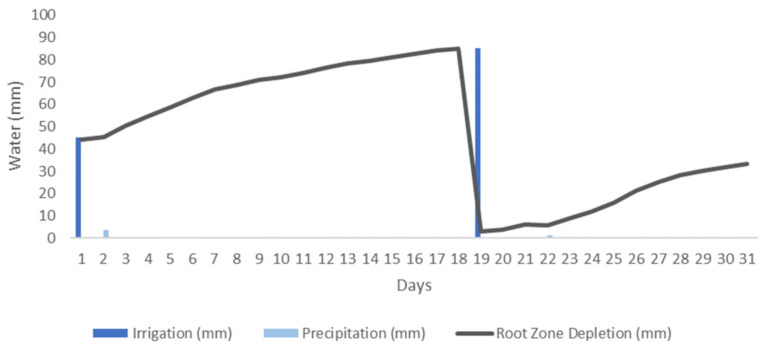
Simulation of an irrigation schedule for an orange field in Gandía.

The case of the irrigation of an orange field in the area of the station of Gandía Marxuquera, Comunidad Valenciana, Spain [57], for the month of October 2020 is presented in Figure 5 as well. In this case, in the area of Gandía, situated in a region further north, there were seven precipitation events. However, four of them were 0.1 mm and thus are not visible on the graph. Considering a first irrigation of 45 mm as in Figure 4, as it can be seen, the next irrigation event is scheduled for day 19 instead, three days after the irrigation event of the MO12 station. In that manner, the algorithm takes into consideration the different weather conditions of each area to provide the necessary amount of irrigation water for each field.

## 3.4. Testbed Description

In this subsection, the testbed for the different deployment strategies is described.

**Figure 6:**
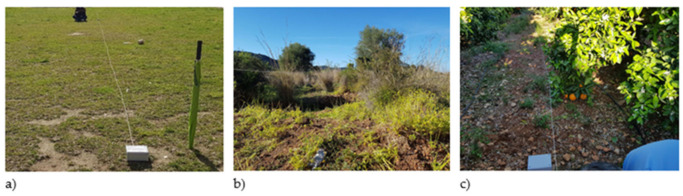
(**a**) Grass field. (**b**) Thicker field. (**c**) Orange field.

The tests were performed at three different types of environments as seen in Figure 6. The first one is a grass field with low vegetation and a regular terrain. The second one is the field with thicket. The terrain of this field presents irregularities in height and a large amount of thicket that obstructs the direct vision between the transmitter and the receiver. The third field is a citrus plot. Orange trees are organized in rows that cover the expanse of the area in an ordered manner. Depending on the position of the emitter and the receiver, trees may or may not obstruct the direct vision between them.

**Figure 7:**
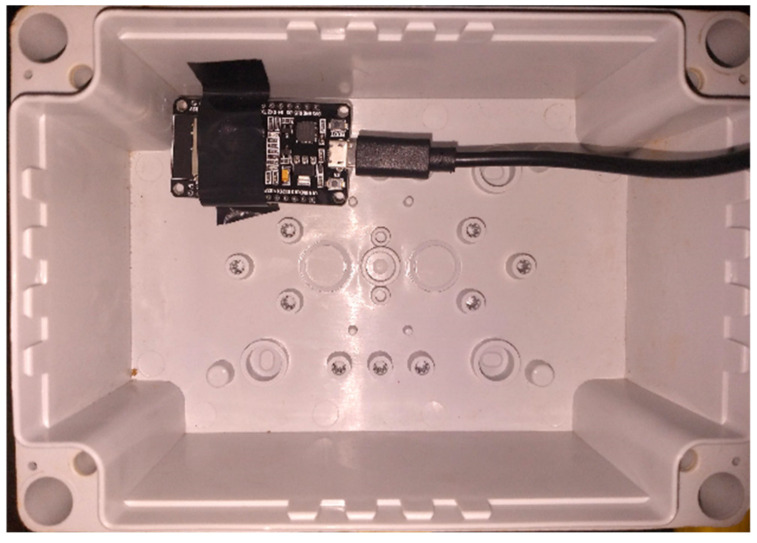
Placement of the node inside the box.

The signal strength was measured utilizing the ESP 32 Devkit v1 programmed utilizing the RSSI function of the Arduino WiFi library and transmitting using the in-built antenna with a time span of 5 s between each transmission. The placement of the nodes inside the protective box can be seen in Figure 7. Five radio transmissions were performed per measurement and spot, and results were averaged to avoid instability in the results as in reference [20].

Two different scenarios were considered, as previously shown in Figure 1. In the first scenario, both transmitter and receiver were placed on the ground to consider the possible disturbance that the nodes may cause if they are positioned at higher heights with some agricultural applications, the machinery, and the usual activities that can be performed by farmers. Furthermore, it is a deployment position that has been considered in soil monitoring scenarios such as [31] that employ drones to collect the data from the on-ground nodes. The aim of testing on-ground deployment strategies with different vegetation types is to determine the feasibility of the communication with nodes placed using this configuration. The measures performed on this scenario were taken with an angle of 0 and 15 degrees to obtain data on how results may change with varied degrees and a variation in vegetation density. Due to the arrangement of the trees in the orange field, it was not possible to make 360-degree measurements, so it was decided to replicate the measurements at 0 and 15 degrees for all vegetated environments in scenario 1. Furthermore, the measures were taken separating the emitter from the receiver, creating more distance between them with each measurement.

**Figure 8:**
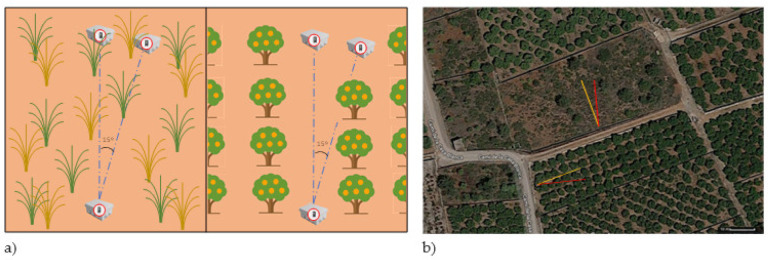
(**a**) Layout of the measures. (**b**) Satellite image of the fields.

In scenario 2, on-ground, near-ground, and above-ground placements of the receiver with varied emitter heights were tested on the citrus plot. The aim of the second scenario is to assess different deployment strategies for crops comprised of trees as opposed to vegetation that presents the bulk of its foliage closer to the ground such as wheat fields [33]. The layout of how the measures were taken is presented in Figure 8a. Due to the differences in the amount of foliage and the structure and layout of the vegetation, the signal strength for each type of vegetation will differ. The satellite image of the fields with vegetation is presented in Figure 8b. It is a Mediterranean area with great extensions of citrus plots and surrounded by mountains. This climate has warm to hot temperatures in summer and cool to warm temperatures in winter, where no below-freezing temperatures are normally reached. Furthermore, the precipitation is low and irregularly distributed. All the measures of each scenario were done on the same day; the temperature remained almost constant at 20 degrees Celsius, as the data were gathered during the central hours of the day. Moreover, there was no presence of precipitations.

## 4. Results

In this section, we show the results of analyzing the received signal strength with different deployment strategies positioning the nodes at several distances in different environments. First, the data gathered in each of the environments (grassland, scrub, and orange field) at two different orientations are evaluated. This way, we study the signal attenuation in the aforementioned environments to show the possible positive and negative effects of having different types of vegetation. Second, we evaluate the signal at different heights of the emitter and receiver at the orange orchard.

### 4.1. Scenario 1: On-Ground Deployment with Different Types of Vegetation

In this subsection, the results of the on-ground deployment strategy with different vegetation environments are presented.

**Figure 9:**
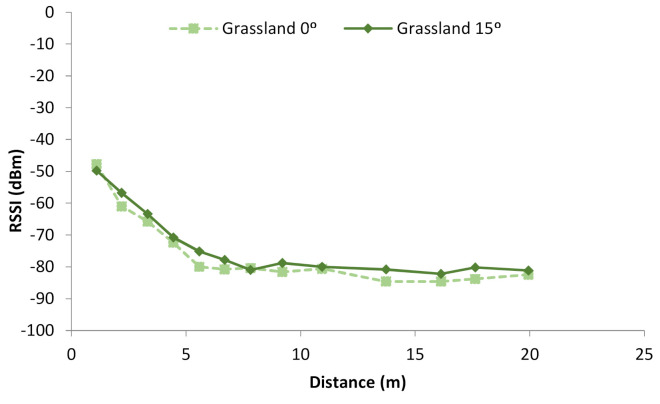
RSSI in grasslands.

Firstly, the results from the on-ground nodes deployed on grasslands are presented. The RSSI of the measures performed at 0° and 15° are shown in Figure 9. As the height of the grass is mostly uniform, the results at both degrees are quite similar. The differences are mainly explained by the irregularities of the terrain and the different irradiation pattern of the antenna. Initially, no objects in the studied area can cause any rebound or reflection of the emitted signal. Thus, we can consider this signal attenuation as the attenuation when both nodes are placed on the soil with no interferences. When the signal is transmitted over grasslands with very low grass, less than 1.5 cm, the RSSI at the maximum measured distance, 20 m, is −81 and −82 dBm at 0° and 15°, respectively. The attenuation is higher in the first 6 to 7 m, decreasing by 30 dBm approximately. Then, the attenuation occurs at a slower pace, and the RSSI values are similar, −80 dBm, until 13 m. The last measured points present lower RSSI, less than −80 dBm.

**Figure 10:**
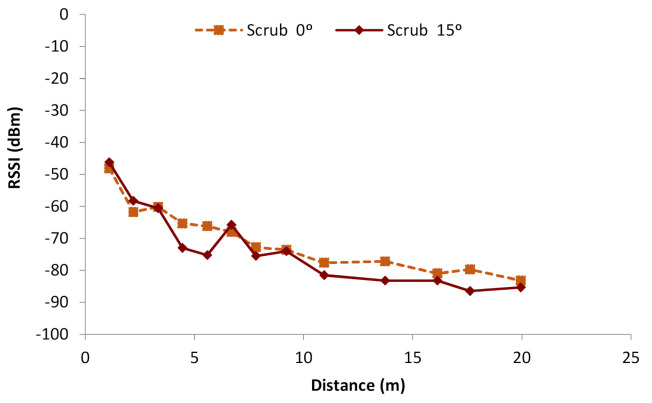
RSSI in scrubs.

The results of the RSSI measures performed in the scrub environment are shown in Figure 10. Initially, as it can be seen, the results from the different orientations show different RSSI values due to the different foliage patterns. As the scrub is not distributed uniformly along the terrain, the RSSI in both orientations is not the same. Furthermore, the data do not present a regular attenuation as in the grassland case. At some points, farther distances present higher RSSI values than those obtained at measurement points situated closer to the emitter. Both observations are mainly explained by the effects of scattering and absorption of this vegetation, which causes shadow areas, and the reflection and refraction might lead to the multipath effect. A clear shadow area can be seen between 4 and 6 m in the case of scrub 15°, where suddenly two points have lower values (−73 and −72.5 dBm). At the maximum distance, the observed RSSI are −83 and −85 dBm. They are notably lower than in the case of grasslands.

Therefore, it is possible to conclude that the shrubs present in the scrub field cause alterations in the attenuation. Furthermore, the multipath effect can be affecting the RSSI received in each measurement greatly, causing the fluctuations in RSSI values. In some cases, for the same measurement point, a variation of more than 10 dBm was found between the maximum and minimum gathered values. Thus, with the data obtained in this study, changes in the vegetation or even some events as blooming, shedding, and growing, or just the wind moving the shrubs, might be affecting the RSSI. Consequently, when the WSN is installed in scrub areas, it is important to note that the RSSI mean values might not be a good indicator, and the minimum values should be considered when making the network planning. Further studies will focus on this aspect.

**Figure 11:**
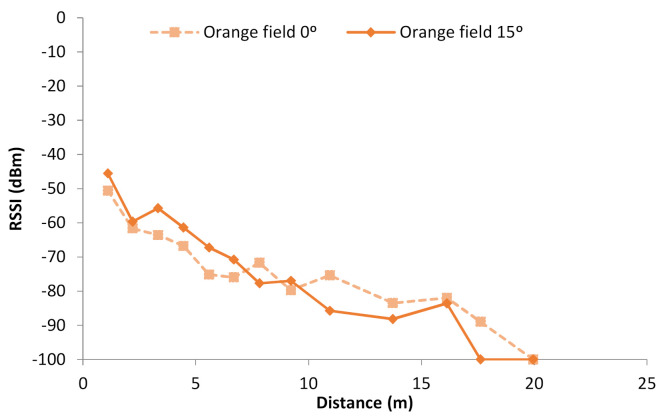
RSSI in Orange fields.

The attenuation of the WiFi signal in the orange fields is now analyzed. As it is explained in the previous section, the data gathered at 0° were taken, having direct vision between the node configured as AP and the node configured as the receiver. On the other hand, the data gathered at 15° were taken without having a direct vision in all the cases, as the trunks of the orange trees obstruct the path between the emitter and the receiver. Figure 11 presents the mean of the RSSI data gathered both at 0° and 15°. We can note that in the first meters, the RSSI is higher in the data gathered at 15° than at 0°. Nonetheless, from 7 m, the RSSI is higher at 0° than at 15°. It is important to note that at 0°, from 16 m, the RSSI starts to decrease very abruptly, losing the connection between 17 and 20 m. In the case of data gathered at 15°, in the last two measured points, there was no connection. In order to represent the data in the graphics, the points where the connection was lost are represented as −100 dBm. 

Thus, in the orange fields, we can conclude that the RSSI of on-ground deployments decreases faster than in grasslands or scrubs. Furthermore, from the obtained data, it is appreciable that, in the first meters, the orange trees may cause constructive interferences at 15°. Nevertheless, at higher distances, the interferences cause negative effects, and the connection is lost earlier than in other environments, and it is lost earlier when there is not direct vision between both devices. This effect will be the object of study for our future works, as this type of crop is abundant in the Mediterranean area, and agriculture systems will be deployed in similar fields.

**Figure 12:**
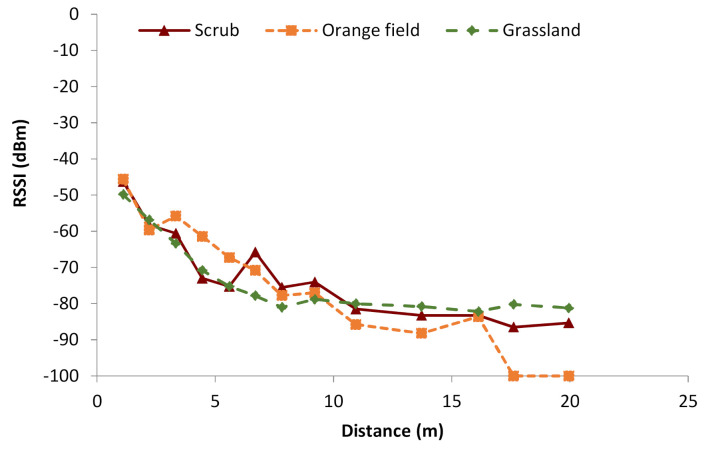
RSSI in all fields.

Finally, a comparison of the data from the grassland, scrub, and orange fields in the same graphics is shown in Figure 12 to determine the differences in the signal attenuation for each type of vegetation. The multipath effect can cause constructive and destructive interferences. As it has been commented on the previous figures, the multipath effect can be considered as the cause of the fluctuations in the measured RSSI values, as there is not only attenuation, but also signal recoveries after obstacles and dense vegetation. From the obtained results, it can be seen that, in the first meters, there is a positive effect on RSSI, with possible constructive interferences that would increment the RSSI. However, after 10 m, there are interferences that cause negative effects. As a result, the RSSI is lower than in environments without vegetation. This can be due to the characteristics of the vegetation. As it can be seen in Figure 6, although the scrubs on the field do present thicker vegetation, there are areas where it is sparser. This type of scrubs and the layout of them is typical of Mediterranean areas. Therefore, there may be areas where the vegetation is not as thick and presents less attenuation. Furthermore, both 0° and 15° measures were similar at many points, so at the position of the sender and receiver, the signal at the scrub field may present elements that introduce less attenuation than that of the grass field. Considering the position of the emitter and receiver at the grass field, the structure of the grass creates a rough surface where the signal is less likely to be reflected, similar to the walls of a small anechoic chamber, whereas the areas with dirt on the scrub field present less vegetation, and as the dirt is compacted, the signal may be reflected. Other studies that perform measures at grasslands and scrublands such as [24] perform their measures in fields with other characteristics. In their case, the scrubland is an area with thick tropical vegetation, and the grass field does not present common grass like that of parks and golf fields. Therefore, as stated before, the characteristics of the vegetation, a thicker, uneven surface in the case of grass and a sparser heterogeneous amount of vegetation in the case of the scrubs, may lead to the obtained results.

After discussing the results obtained from the RSSI measures of the on-ground deployment strategy in different vegetation environments, a model of the signal quality for each vegetation will be obtained to determine the theoretical maximum coverage for the on-ground deployment. 

Equation (1) describes the power balance formula that determines the received signal power (Prx) according to the transmitted power, the gain of both the transmitter and receiver antennas, and the losses from air transmission, humidity, and vegetation as in [44]. However, unlike in [44], the signal loss produced by the vegetation, in this case, is not that of [58], but will be obtained from the real measures performed in different environments.

P_rx_(dB) = P_tx-1m_(dB) − 10n log d (m) − L_humidity_(dB) − L_vegetation_(dB) (1)

Most of the values are known, such as the transmitted power (P_tx-1m_) that for the ESP32 is −45.75 dBm, which can be theoretically calculated through Equation (2), where the frequency is expressed in Hz and c is 3 × 10^8^ m/s. The characteristics of the ESP32 chip are depicted in [52]. When the medium is air, the value n equals 2. The distance between transmitter and receiver is d. Lastly, L_humidity_ is 0.026 dB for the hydrometric H and K areas of Spain [59].

P_tx-1m_ = 20 log_10_ d + 20 log_10_(f) + 20 log_10_(4π/c) (2)

In order to determine the signal loss caused by each type of vegetation, the mathematical model for the P_rx_ for each type of vegetation needs to be obtained.

**Figure 13:**
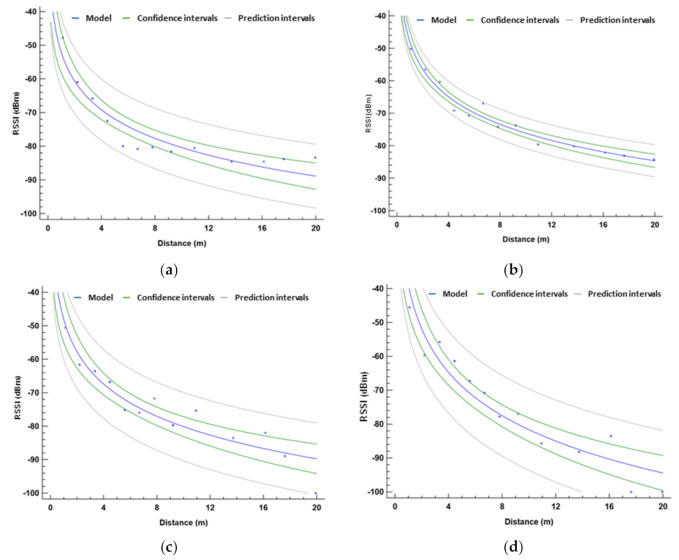
Model of RSSI in (**a**) grasslands, (**b**) scrubs, (**c**) orange fields at 0°, and (**d**) orange fields at 15°.

The data from Figure 9 can be adjusted to a mathematical model, which will be our signal attenuation model with no interferences. As the observed differences at the same distance but with different orientations are minimum, we are going to consider that the RSSI does not change with the orientation of the antenna. We have observed that this happens with low vegetation environments in contrast to high vegetation environments where the results may vary with different angles. Thus, for the case of the grasslands, both sets of data are to be used for the mathematical model, see Figure 13a. The mathematical model is a logarithmic model, and its expression can be seen in Equation (3). In Figure 13, the gathered data are represented in squares and the lines represent the model, its confidence intervals, and its prediction intervals. The correlation coefficient of this model is 0.98, the R2 is 96.68%, and the mean absolute error is 1.44.

P_rx-grass_ (dBm) = −52.25 − 12.24 ln d(m) (3)

As in the grassland, we can obtain a mathematical equation that models the gathered data. As in the previous case, the mean of both sets of data is used. The obtained model follows the same pattern as the previous one and is shown in Equation (4). The mathematical model, with the intervals of confidence and prediction, can be seen in Figure 13b. The correlation coefficient of this model is 0.93, the R2 is 87.86%, and the mean absolute error is 3.40.

P_rx-scrub_ (dBm) = −47.95 − 12.25 ln d(m) (4)

Regarding the orange fields, as we consider that the interferences are different at 0° and 15°, the gathered data are going to be utilized separately to build two mathematical models. The first one would be for a deployment strategy where the nodes are located at the line of sight, placed on the streets between the rows of trees. The mean of the data gathered at 0° is utilized to create a model, see Equation (5) and Figure 13c. The correlation coefficient of this model is 0.94, the R2 is 88.84%, and the mean absolute error is 3.10.

P_rx-orange 0°_ (dBm) = −48.15 − 13.89 ln d(m) (5)

Secondly, the model for the deployment where the trajectory between the emitter and receiver is obstructed by the tree trunks is obtained. The model from the data gathered at 15° is presented in Equation (6) and Figure 13d. The correlation coefficient of this model is 0.95, the R2 is 91.08%, and the mean absolute error is 4.29. We can compare both models and see that they are similar, but the slope is more pronounced in the second case.

P_rx-orange 15°_ (dBm) = −39.12 − 18.74 ln d(m) (6)

From the obtained models, the signal loss caused by each type of vegetation can be obtained. Therefore, the theoretical equation and the model are equivalent, as shown in Equation (7), which is obtained for the grass mathematical model.

P_tx_ − 10n log d − L_rain_ − L_grass_ = −52.25 − 12.24 ln d (7)

Therefore, the signal loss caused by grass can be expressed as Equation (8).

L_grass_ = P_tx_ − 10n log d − L_rain_ + 52.25 +12.24 ln d (8)

When the known values are replaced in Equation (8), the resulting equation for the signal loss caused by grass is Equation (9).

L_grass_ = 6.474 − 20 log d + 12.21 ln d (9)

Equation (9) can be then simplified to Equation (10).

L_grass_ = 3.554 ln d + 6.474 (10)

Following the same process, the resulting equations for the rest of the vegetation types are Equation (11) for scrub, Equation (12) for the orange trees at 0 degrees, and Equation (13) for the orange trees at 15 degrees.

L_scrub_ = 3.5641 ln d + 2.174 (11)

L_orange_tree_0_ = 5.2041 ln d + 2.374 (12)

L_orange_tree_15_ = 10.0541 ln d − 6.656 (13)

**Figure 14:**
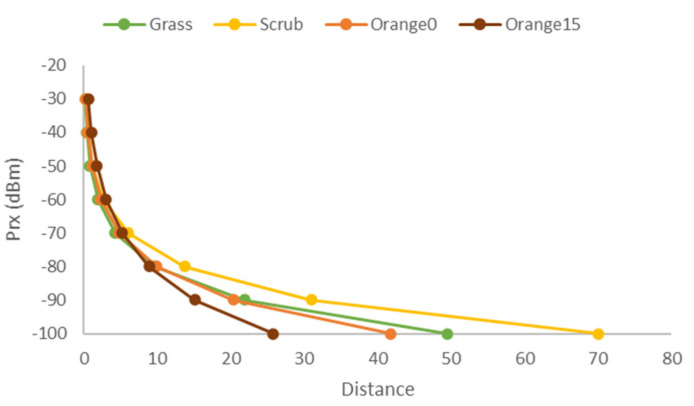
Maximum distance for desired Prx.

In other to determine the maximum distance between nodes for each type of vegetation, the aforementioned expressions for signal loss for each type of vegetation should be applied to Equation (14).

d = 10^(Ptx − Lrain − Lvegetation − Prx)/20^ (14)

where L_vegetation_ is replaced by L_grass_, L_scrub_, L_orange0,_ and L_orange15_ accordingly. The results for the maximum distance for each type of vegetation can be seen in Figure 14. Although results show distances greater than those of the empirical data, the real data for RSSI values between −90 and −100 are harder to obtain due to the nodes not connecting to each other. However, the model does not regard that. However, as it can be seen, for the orange field, values above −90 stay within the 20 m limit. Considering a signal strength between −90 and −100 is of bad quality and would not be considered when designing the network, values are reflecting the empirical case.

### 4.2. Scenario 2: On-Ground, Near-Ground, and Above-Ground Deployments for Orange Tree Monitoring

In this subsection, the results for the scenario with on-ground, near-ground, and above-ground deployment strategies with different emitter heights at an orange orchard are presented.

In this scenario, the results have been classified according to the height of the receiver, as there was more variability with changes in the height of the receiver than with changes in the height of the emitter. The different receiver heights that were measured were on-ground (0 m), near-ground (0.5 m), and above-ground (1 m). While the different heights of the emitter were 0.5, 1, 1.5, and 2 m. 

**Figure 15:**
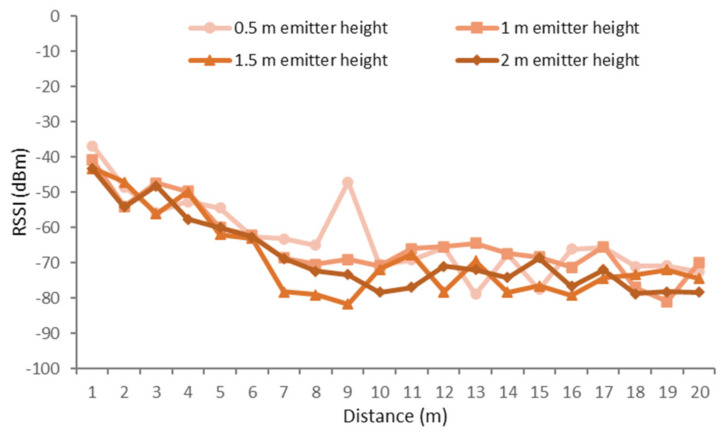
RSSI for on-ground receiver and different configurations for the emitter.

The results for the receiver on the on-ground position and different positions of the emitter are presented in Figure 15. As it can be seen, the higher signal qualities were obtained for the emitter height of 50 cm for most distances. Good results were obtained as well for the height of 1 m. The lower RSSI values obtained at a height of 1.5 and 2 m are due to a high density of the foliage of the trees. The average height of the orange trees was 2.5 m. Therefore, the bulk of the foliage was between 1 and 2.5 m. That led to great interferences for those heights when a tree was reached. It can be seen as well how the RSSI fluctuates at different distances alternating between lower and higher RSSI values. This effect can be due to the multipath effect as a result of the reflection on leaves and fruits as well as the attenuation caused by the foliage. One of these fluctuations is the peak reached at the 0.5-m height for a distance of 9 m at an area with abundant foliage. For the 0.5-m case, the signal is boosted; however, for the rest of the emitter heights, the quality of the signal was reduced as the emitter was placed among the foliage. It is noticeable as well how the quality of the signal is recovered after an area with abundant foliage.

**Figure 16:**
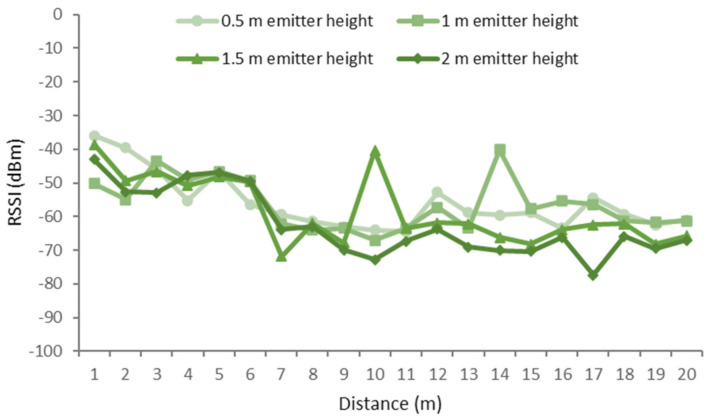
RSSI for near-ground receiver and different configurations for the emitter.

For the near-ground configuration of the height of the receiver (0.5 m), the results showed in Figure 16 were obtained. In this case, the best RSSI values were obtained for the emitter at a height of 1 m. The overall signal quality is better than that of the on-ground position of the receiver. However, the signal quality is less stable, resulting in high fluctuations along the different measurement spots as the distance between emitter and receiver increases. Particularly, the fluctuations of the RSSI at different heights, compared to those of the on-ground receiver position shown in Figure 15, are more evident with acute fluctuations of 27.4 dBm at the 1.5 m emitter height and 23.24 dBm at the 1 m emitter height. The lowest RSSI values were in the range between −80 and −70 dBm for the 1.5 m height. These values were due to a highly dense mass of foliage from the top of the orange trees. However, when the highly dense foliage is surpassed, the signal is recovered. Particularly, after the low values reached at meter 17 for the emitter height of 2 m, the signal was recovered in 11.5 dBm. 

**Figure 17:**
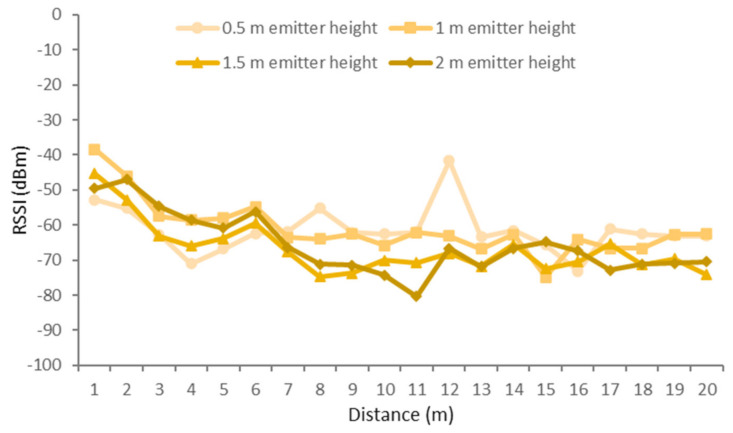
RSSI for above-ground receiver and different configurations for the emitter.

Lastly, Figure 17 presents the RSSI values for the receiver at the above-ground position (1 m) and the emitter at different heights and distances. This configuration presents worse signal quality than that of Figure 15 and Figure 16, as the line of sight between the emitter and receiver nodes is at the same height as the bulk of the foliage. In the case of the above-ground position of the receiver, the emitter heights of 1 and 0.5 m are the best options, as the RSSI values indicate better signal quality than those of the emitter heights of 1.5 and 2 m. This is due to the attenuation caused by the highly dense foliage of the treetops that obstructed the line of sight between emitter and receiver. As the signal was attenuated by a more constant density of foliage, the fluctuations in signal quality are in most cases less prominent than those of the on-ground and near-ground deployments. However, for the emitter height of 0.5 m, as it was not obstructed with dense foliage, there are great variations with several peaks. The first peak presents a degradation of the signal, which reaches a low value of −71 dBm at a distance of 4 m to the receiver. Then, the signal recovers and reaches a peak with a high value of −55.16 dBm at a distance of 8 m from the receiver. Then, another high peak is reached with a value of −41.66 dBm at a distance of 12 m from the receiver, which is a fluctuation of 20.34 dBm. Lastly, another low peak happens at a distance of 16 m from the receiver with a value of −73.16 dBm.

Considering all the receiver deployment strategies, it can be seen that for emitter heights of 0.5 and 1 m, with no obstruction of the line of sight between emitter and receiver, the signal presents high fluctuations that may be caused by the multipath effect. For the case of the 1 m emitter heights, the fluctuations are less prominent than for the case of 0.5 m with similar RSSI values at the points with no peaks. Therefore, due to the stability of the signal and the good RSSI values, the emitter height of 1 m is the best option for all on-ground, near-ground, and above-ground receiver positions. While emitter heights of 1.5 and 2 m are more stable, they are also more affected by the attenuation of the highly dense foliage, as it is to be expected. Thus, the obtained RSSI values are generally lower than those of the 0.5 and 1 m emitter heights.

**Figure 18:**
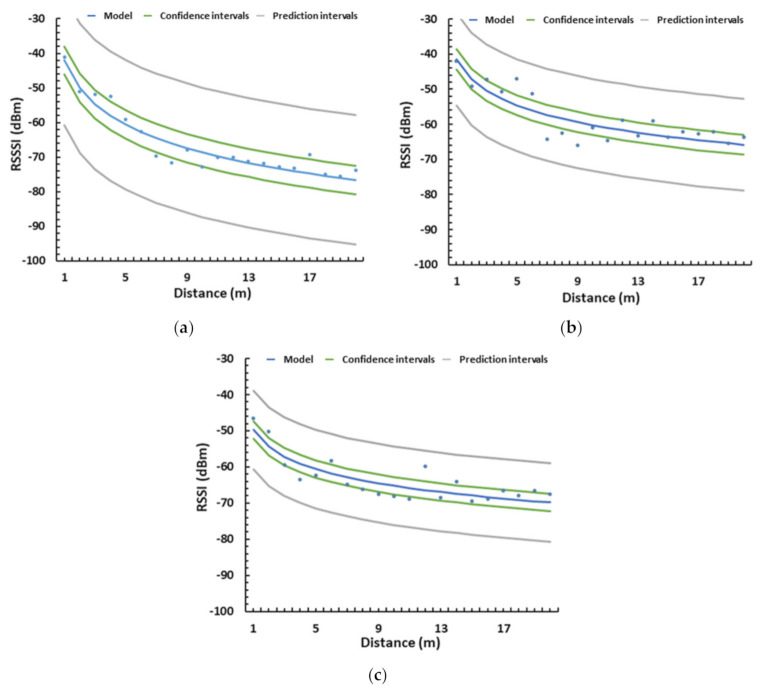
Model of RSSI in the orange field with emitter (**a**) on the ground (**b**) at 50 cm of height (**c**) at 1 m of height.

From the obtained signal quality results, a heuristic signal attenuation model was obtained for the on-ground, near-ground, and above-ground deployments after discarding the outlier values. As in the first scenario, the aim to obtain a theoretical maximum coverage to aid in the design of a soil sensing network deployment. The model presented in Equation (15) was obtained for the on-ground deployment. In Figure 18a, the data gathered from the tests performed in real environments are presented as dots. The model, confidence intervals, and prediction intervals are provided as well. For the near-ground deployment, the model presented in Figure 18b is expressed as Equation (16). As it can be seen, this is the configuration with better signal quality. Lastly, the model for the above-ground deployment is presented in Equation (17). The graphic representation of the model, the confidence, and prediction intervals are shown in Figure 18c.

P_rx-on-ground_ (dBm) = −42.05 − 11.53 ln d(m) (15)

P_rx-near-ground_ (dBm) = −41.55 − 8.10 ln d(m) (16)

P_rx-above-ground_ (dBm) = −49.80 − 6.68 ln d(m) (17)

Utilizing Equation (1) and the models presented in Equations (15)–(17), the vegetation losses caused by the foliage of the trees at the on-ground, near-ground, and above-ground deployments are presented in Equations (18)–(20), respectively.

L_veg_on_gorund_ = 20.22 ln d + 12.03 (18)

L_veg_near_ground_ = 16.79 ln d − 2.68 (19)

L_veg_above_ground_ = 15.37 ln d + 17.18 (20)

**Figure 19:**
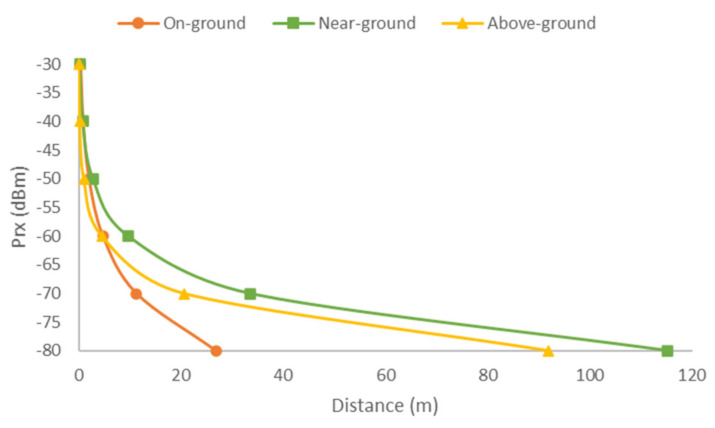
Maximum distance for desired Prx at on-ground, near-ground, and above-ground deployments.

Lastly, applying Equation (14), the maximum theoretical distance for a determined Prx is obtained (See Figure 19), where L_vegetation_ is replaced by L_veg_on_gorund_, Lveg__near_ground_, and L_veg_above_ground_. Acceptable signal quality would be obtained up to 26 m for the on-ground deployments, 115 m for the near-ground deployments, and 91 m for the above-ground deployments. Considering these results, it can be concluded that near-ground deployment strategies are the best option for soil monitoring network deployments in crops comprised of trees, such as orange orchards. The 0.5 and 1 m height were found to be the better option for emitter positioning, which could be expected, as it is a height with a low presence of foliage. However, even with no or few obstructions of the line of sight between emitter and receiver, the signal quality presented high fluctuations, which were less acute for the case of the emitter placed at a height of 1 m.

## 5. Discussion and Challenges

In this section, a discussion on transmitting at different heights in environments with vegetations is provided. Furthermore, the challenges of deploying wireless sensor networks for precision agriculture are presented.

Node height is one of the key factors of sensor network deployments in rural and vegetated areas due to multiple reasons. Coverage and signal quality are one of these reasons, as the main objective of the sensor network deployment is to gather and forward the data so they can be accessed by the user. However, other aspects such as the amount of foliage [22,26,27], the plant height [26], the machinery utilized at the fields, the presence of animals, or the irrigation system may present some challenges. Thus, the aim when designing the sensor network deployment for a determined rural environment is to consider the specific challenges and needs of the area while opting for the best signal quality and coverage configuration. The need of performing preliminary tests to determine the real performance of the nodes in contrast to the advertised by the manufacturer was also stressed in other studies [33], with an emphasis on low-cost nodes such as the ones utilized in our testbed. As there are many variables to consider, other testbeds have focused on specific environments, node height, and frequencies to study how the signal is affected (see Table 1).

The crop type is always described on the testbeds presented in Table 1, as it is an indicator of the plant height and possible foliage density. Particularly, the effects of the attenuation caused by high foliage density were remarked upon in [21]. Though many studies have been performed regarding the effects of vegetation on signal quality, each study provides new insights on a specific type of crop, wireless technology, the response of a specific node, or the performance of the deployment strategy, among other variables. Furthermore, a useful and interesting use of monitoring signal quality in vegetated environments is to use RSSI to identify vegetation growth [33]. Nonetheless, the deployment strategy is to be considered, as the height of the node that is measuring the signal quality should be known to establish the reference height value of the crop. Crops such as lettuce or potatoes [29] have a low height, present a uniform foliage density for the height of the plant, and are planted with a small distance between plants. Thus, on-ground deployments would not be suitable for the characteristics of this type of crop. Asparagus, like some types of shrubs [23], present a medium height, the foliage density is uniform as well, and the separation between plants can be greater between rows. In this case, on-ground deployments would be possible if the nodes are deployed between rows. However, as the distance between rows is small, it could be an inconvenience for the farmers to access the plants. Therefore, above-ground deployments with posts placed on the plant row would be the best solution. Lastly, plants with high heights such as orange trees are planted with a separation between 3 and 5 m and can reach heights above 7 m. The bulk of the foliage is at the treetop, with space without foliage at the tree trunk level. Therefore, above-ground deployments would be susceptible to more signal interferences and would interfere with farming activities such as pruning. Furthermore, considering the results of the presented testbed, a near-ground deployment strategy would be the best option. However, the results show a high fluctuation of the signal quality at emitter heights of 0.5 and 1 m, with no obstructions in the line of sight between emitter and receiver that can be caused by the multipath effect.

The node density is another aspect to consider. Deploying one node per plant is neither necessary nor affordable. The data that are necessary to calculate irrigation needs or other aspects such as fertilizer needs do not vary significantly from plant to plant. Therefore, the field can be divided into different zones with a node or a cluster of nodes per zone. Moreover, the cost of deploying nodes all over the field can be too elevated for most of the farmers. As such, the nodes can be deployed as clusters that monitor one area and communicate with an AP or gateway that forwards the data to the user or the data center. Furthermore, if the nodes in the cluster are utilized to detect false positives and false negatives or to evaluate the area reached by the water in the case of drip irrigation, the distance between the nodes will not be very high as well. Lastly, as meteorology data are also necessary for the calculation of the irrigation needs, and the data cannot be measured near the ground, if a node for meteorology monitoring is utilized, the node should be deployed above-ground and in an exposed area as it is recommended by the FAO [54].

The type of irrigation may also influence the decision-making process when designing a WSN deployment for a field. Flood irrigation would make on-ground deployments impossible, as the nodes could get damaged or could move even when placed in protective boxes. For pivot irrigation, the node cannot be placed at a height higher than the shortest point of the structure of the irrigation system. Furthermore, for drip irrigation, wet areas can be avoided, which facilitates the deployment of the nodes.

Energy consumption is a recurrent topic regarding WSN. Although the amount of daily data necessary for irrigation calculations can be as few as one value for each variable, most systems will gather data in real-time or at frequencies of 10 min, 30 min, or 1 h. Therefore, energy harvesting solutions such as the use of solar panels have been widely implemented [60]. Furthermore, the performance of the energy harvesting solutions can be monitored by the nodes to ensure there is enough energy [61]. However, the foliage of the plants could create a shade on the panel resulting in a reduction or the absence of harvested energy. Therefore, this aspect should also be considered when selecting the height of the node. There are however new solutions that allow charging the batteries of the nodes in a fast manner without the need of connecting the nodes to any device or removing the insulation, such as utilizing wireless power transfer (WPT) [62].

Finally, the selection of the wireless technology to be used on the precision monitoring system is also to be considered. Aspects such as coverage distance, data rate, energy consumption, equipment cost, and the simplicity of implementation are often evaluated when taking this decision. Regarding coverage, there are low range technologies such as Bluetooth, mid-range technologies such as Zigbee or Wi-Fi, and long-range technologies such as LoRa. High data rates are not often necessary for PA monitoring systems; therefore, most technologies could be utilized. Regarding the cost of the devices, WiFi has been the technology with the most options regarding price ranges. However, ZigBee and LoRa devices have had a cost reduction in recent years, making them more affordable. Lastly, Wi-Fi is the easiest technology to use regarding implementation simplicity due to both the available options in the market and the extensive documentation, removing in many cases the need for an expert to deploy the network. This accessibility results in this technology being significantly the most utilized in IoT systems for PA [60], followed by GSM and ZigBee. In terms of energy consumption, Bluetooth Low Energy (BLE), ZigBee, or LoRa are presented as low-power technologies. Among these technologies, ZigBee is the most used low-power technology in agriculture networks [60], and its performance in vegetated environments has been studied in detail [33]. WiFi presents higher energy consumption. However, for Ad Hoc networks, WiFi was found to have more energy efficiency than LoRa in shorter distances [63], as LoRa is more efficient for distances greater than 300 m. Furthermore, as it was commented previously, energy harvesting solutions help in alleviating the higher energy consumption of non-low-power technologies. It could be argued that ZigBee would be the best technology for PA systems, and it is the best option regarding energy consumption, but the convenience of Wi-Fi often wins in deciding the wireless technology if the requirements for coverage and energy consumption are met, which it does for many architectures and the few data that need to be forwarded. Furthermore, Wi-Fi is convenient for the user as well if there is a need to connect directly with a device utilizing a smartphone, tablet, or laptop, which is the case for remote areas, and countries without a well-developed communication infrastructure.

### Limitations of This Study

Precision agriculture systems have specific needs that require the consideration of specific aspects of the agricultural environment to be monitored. This paper provides a first approach to identify the aspects that affect the deployment of wireless networks for precision agriculture systems. It is thus limited to the specific types of vegetation that were considered in the testbed and to a specific low-cost ESP32 WiFi node. Furthermore, this study is limited to sunny weather conditions with medium temperatures, which is the predominant weather on the Mediterranean coast of Spain. Although similar results are to be expected with a replication of this testbed utilizing low-cost WiFi nodes, more tests and replications are needed to determine the exact influence of certain aspects such as weather conditions, different stages of the crops where there is presence of flowers and fruits, and the use other types of low-cost nodes to determine if there are significant differences among them. However, although this study provides a first approach to perform soil monitoring wireless node deployments, we consider that our study provides some insights to wireless sensor deployments in orange fields that could be of interest to those interested in deploying a precision agriculture system to monitor citrus crops or other types of tree crops.

## 6. Conclusions

The implementation of IoT systems in rural areas may present some challenges due to the vegetation. Particularly, soil monitoring agriculture applications may suffer from the interferences caused by the density of the foliage or some characteristics of the plants such as height or width. In this paper, we have proposed a soil monitoring system comprised of a soil humidity multi-sensor array, a soil temperature sensor, and a pH sensor, and we have performed a study on fields with different deployment configurations with varied types of vegetation. Furthermore, on-ground, near-ground, and above-ground node placements were tested as well. Results have shown that vegetation creates a high variability in areas with high foliage density. The maximum theoretical coverage was obtained for each configuration. On-ground deployments had the least coverage even with vegetation where the bulk of the foliage is at higher heights. On the other hand, near-ground deployments provide the best coverage with orange trees. However, on-ground and near-ground deployment strategies in orange fields presented high variability of signal quality even with no obstacles in the line of sight between emitter and receiver, as opposed to higher emitter heights that presented a more attenuated but stable signal quality. Nonetheless, the aspects of the rural environment and the deployment that affect the signal such as node height, crop type, foliage density, or the form of irrigation must be considered when designing a WSN deployment for PA systems as it has been discussed.

For future work, we will perform a deployment study for other areas monitored in PA systems such as the canals where the irrigation water is transported to determine the factors that affect the communication in those environments.

## Figures and Tables

**Table 1:** Comparative of testbeds of studies on the effects of vegetation on the wireless signal.

Ref	Environments	Heights	Frequency	Transmitter	Receiver
Jose Antonio Gay-Fernández et al. [21]	Forests, scrublands, and grasslands	0.9, 1.2, and 1.6 m	2.4, 3.5, and 5.5 GHz	Rhode-Schwarz SMR-40 signal generator and an Electronics EM 6865 wideband antenna	Robde-Schwarz FSH-6 spectrum analyzer
Muhammad A. et al. [20]	Urban areas, rural areas, forests, and plantations	-	2.4 GHz	-	-
Hairani Maisarah Rahim et al. [22]	Tropical vegetation foliage	2 m	2–18 GHz and 26.5–40 GHz	Anritsu MG3694C Signal Generator	Spectrum Master MS2730T
J. Acuña et al. [23]	Shrubs	1.25 m	2.4 and 5.8 GHz	Rohde & Schwarz radio signal generator SMR-40	Rhode & Schwarz FSP-40 spectrum analyzer
Leire Azpilicueta et al. [24]	Park	1 m	2.4 GHz	Zigbee mote	Agilent N9912 Field Fox portable spectrum analyzer
Jürgen Richter et al. [25]	Trees	5–17 m	1–60 GHz	-	-
Nick Savage et al. [28]	Trees	2.5–7.5 m	1.3, 2, and 11.6 GHz	Channel sounder	Channel sounder
John Thelen et al. [29]	Potato crop	0 m	433 MHz	Mica2Dot	Mica2 with MIB510 programming board and an antenna with an 11.7 dB gain
B. Dhanavanthan et al. [34]	Cornfields and coconut gardens.	2 cm, 15 cm, and 1 m	2.4 GHz	Agilent N5182A Vector Signal Generator	Agilent N9010A Vector Signal Analyzer
Andrew Szajna et al. [35]	Sports facility and a forested area covered by snow.	0–130.8 cm	2.45 GHz	National Instruments PXI-5670	National Instruments PXI-5690 low noise preamplifier paired with National Instruments PXI-5660 RF vector signal analyzer.
Daihua Wang et al. [36]	A plaza, a sidewalk, and a grassland.	3 cm, 1 m	2.4 GHz	RF transceiver working at 2.4GHz	Agilent N9912A spectrum analyzer
Weisheng Tang et al. [37]	Concrete road, flat grass, and undulating grass.	5 cm, 50 cm, and 1 m	470 MHz	Silicon Labs Si4432 radio frequency chip	MSP430F5438 as the Microprogramed Control Unit (MCU) chip
Seun Sangodoyin et al. [38]	Rural flat and hilly terrains.	10 cm, 20 cm, 50 cm, and 2 m	3–10 GHz	Tektronix AWG 7122c waveform generator	Agilent DSA91304A Digital Sampling Oscilloscope
Amir Torabi et al. [39]	Ground plain, yard, and grass park.	13 cm	315 MHz, 915 MHz, 2.4 GHz	-	-
Daniel P. Luciani et al. [40]	Concrete, grass field and hallway.	15 cm, 30 cm, and 1 m	2.48 GHz	STMicroelectronics STM32W-RFCKIT	Laptop
Hicham Klaina et al. [41]	Soil, short and tall grass fields	20 and 40 cm	868 MHz, 2.4 GHz, and 5.8 GHz	ZigBee nodes	ZigBee nodes
Peio Lopez-Iturri et al. [42]	Oak and pine tree fields.	1, 2, and 3 m	2.4 GHz	-	-
D. L. Ndzi et al. [43]	Mango and palm plantations.	1.3, 1.7, 2.2, and 2.6 m	0.4–7.2 GHz	Agilent E8267D signal generator	Agilent E4405B spectrum analyzer
Jaime Lloret et al. [44]	Rural and forest areas.	3 m	2.412–2.472 GHz	wireless multisensors and wireless IP cameras	802.11g access points
Our testbed	Orange field, scrubland, and grassland	0, 0.5, 1 m	2.4 GHz	ESP 32 Doit devkit v1	ESP 32 Doit devkit v1

**Table 2:** Variables required by the system.

**Fixed Variables**	**Variables Set by the User**
Elevation above sea level	Height of the tree
Date	Selection of soil type
Latitude	Time period for irrigation calculation
Height of wind speed measurement	Selection of Single Coefficient Approach or Dual Coefficient Approach for ETc calculation
**Variables Obtained from the Monitored Data**	
Maximum air temperature of the day	Water salinity
Minimum air temperature of the day	Soil conductivity
Maximum relative humidity of the day	Soil humidity
Minimum relative humidity of the day	Soil temperature
Hours of sunlight of the day	Mean temperature of the actual month
Wind speed	Mean temperature of the previous month
Precipitation amount	Estimated mean temperature of the following month
Hour of the precipitation	

## Data Availability

The data presented in this study are available on request from the corresponding author. The data are not publicly available due to privacy constrains.
